# Insights into Controlling the Spread of COVID-19: A Study Inspired by Seven of the Earliest Vaccinated Countries

**DOI:** 10.1155/2022/4533957

**Published:** 2022-09-20

**Authors:** Jane K. L. Teh, David A. Bradley, Woo Teck Ang, Kok Lay Teo, Jack Bee Chook, Kee Huong Lai, Suat-Cheng Peh

**Affiliations:** ^1^School of Mathematical Sciences, Sunway University, 47500 Selangor, Malaysia; ^2^Healthy Ageing and Well-Being Research Cluster, Sunway University, Malaysia; ^3^School of Engineering and Technology, Sunway University, 47500 Selangor, Malaysia; ^4^PEMANDU Associates Sdn Bhd, Sunway Putra Tower, 50350 Kuala Lumpur, Malaysia; ^5^School of Medical and Life Sciences, Sunway University, 47500 Selangor, Malaysia

## Abstract

**Background:**

The aim of the study is to derive deeper insights into the control of the spread of COVID-19 during the second half of 2021, from seven countries that are among the earliest to have accelerated the deployment of COVID-19 vaccines. *Methodology*. This study used data from the Global COVID-19 Index and Google COVID-19 Community Mobility Reports. Data was extracted on the 5^th^ of each month from July to December 2021. Seven countries were selected—United Kingdom, United States of America, Israel, Canada, France, Italy, and Austria. The sample comprised number of new cases, hospitalisations, ICU admissions and deaths due to COVID-19, government stringency measures, partial and full vaccination coverage, and changes in human mobility. Principal component analysis was conducted, and the results were interpreted and visualized through 2-dimensional and 3-dimensional plots to reveal the systematic patterns of the data.

**Results:**

The first three principal components captured around 77.3% of variance in the data. The first component was driven by the spread of COVID-19 (31.6%), the second by mobility activities (transit, retail, and recreational) (24.3%), whereas the third by vaccination coverage, workplace-related mobility, and government stringency measures (21.4%). Visualizations showed lower or moderate levels of severity in COVID-19 during this period for most countries. By contrast, the surge in the USA was more severe especially in September 2021. Human mobility activities peaked in September for most countries and then receded in the following months as more stringent government measures were imposed, and countries began to grapple with a surge in COVID-19 cases.

**Conclusion:**

This study delineated the spread of COVID-19, human mobility patterns, widespread vaccination coverage, and government stringency measures on the overall control of COVID-19. While at least moderate levels of stringency measures are needed, high vaccine coverage is particularly important in curbing the spread of this disease.

## 1. Introduction

It is evident that the COVID-19 pandemic has brought about a global health crisis with massive premature loss of life [1, 2]. The pandemic has also resulted in severe economic consequences all over the world and has led to disruptions in business and other industries [3–5]. In reactive responses, countries are applying various policy measures, seeking to contain the COVID-19 pandemic, and making a move towards reopening economies. Among the major policy interventions adopted by many developed countries around the world, accelerating vaccination coverage in their respective countries has been a focus [6, 7].

Notable in the rapid conduct of mass vaccination, not to the exclusion of a number of other example nations, have been the United Kingdom, several other countries from the European continent, North America, and also Israel [6, 8, 9]. Within these examples, there have been those nations that experienced some of the earliest surges in the COVID-19 pandemic, rapidly followed by associated devastating consequences, a situation with which the entire world has since become only too familiar [10, 11]. In those same example countries, there are signs of a turnaround, most markedly towards the middle of 2021, benefitting from the swift development of vaccines, access to these in meaningful amounts, and an ability to harness capability within the national health system for the conduct of mass vaccination. While the recovery is not entirely clear from the statistics of the number of cases, the evidence is certainly evident from the reduction in severe outcomes, hospitalizations, and deaths as seen in the USA and UK [12–16].

Indubitably with mass vaccination rollout, the easing of restrictions together with more lenient business reopening policies has been instrumental in reviving the labour market [17, 18]. Several studies have proposed strategies at which restrictions can be lifted alongside mass vaccination [19, 20], as well as vaccination rollout strategies targeting specific groups of people in reducing the social distancing mandate [21, 22]. Parallel to this, the magnitude of human mobility has increased substantially, especially in countries with higher levels of Human Development Index and labour force participation [23].

Concomitantly during the second half of 2021, the COVID-19 Delta variant of concern, which was first identified in India in December 2020, has emerged as the dominant strain across much of the European region and caused concerns over Europe's economic rebound [24–26]. The Delta variant has been found to be 40–60% more transmissible than the Alpha variant and leads to an increased risk in hospitalizations, ICU admissions, and deaths [27, 28]. Despite this, the pandemic has been perceived to be largely controlled, alongside widespread vaccine coverage in the UK, Israel, and parts of Europe [6, 7, 26]. Those who have been vaccinated were much less likely to end up in hospital and were protected from developing severe disease, hospitalization, and death due to the Delta variant [16, 29, 30]. However, inferential analysis studying the effects on vaccination on the Delta variant has implied that the effects of vaccination decrease over time [31, 32]. Moreover, the highly transmissible Delta variant has caused resurgences of infections, even in areas with high vaccination coverage. As booster vaccinations may help to control transmission, several countries such as Israel and the UK have started administering COVID-19 vaccine boosters, but to targeted population groups during the second half of 2021 [16, 30, 33].

Many events have unfolded during the second half of 2021 in many of these developed countries—widespread mass COVID-19 vaccination; the easing of government restrictions which led to reopening of economies and increase in human mobility; and a surge in COVID-19 cases as the deadly and highly transmissible Delta variant became the dominant strain. Therefore, deeper insights are needed on the overall state of the COVID-19 pandemic, when all of these confounding factors are taken into consideration. Moreover, there is scarcity in research of a similar pandemic period, where all of these factors are examined simultaneously. Hence, we attempt to examine, through an unsupervised machine learning approach of the following dimensions during the second half of 2021, the spread of COVID-19, mass vaccination rollout, stringency measures by governments, and human mobility patterns. During this period, Delta is the dominant strain, while Omicron is yet evident.

## 2. Methods

In this section, we describe the dataset of the study, the principal component analysis technique as a suitable tool to achieve the objectives of this study, and the analytical procedures.

### 2.1. Dataset

#### 2.1.1. Global COVID-19 Index

We have acquired access to data from the Global COVID-19 Index (GCI) provided by PEMANDU Associates [34]. The GCI is an independent data engine covering 180 economies and consolidates to provide open data. This includes the daily number of COVID-19 cases, deaths, recoveries, hospitalisations, and vaccination from the COVID-19 Data Repositories by the World Health Organisation, Our World in Data, Johns Hopkins Center for Systems Science and Engineering (JHU CSSE) and Oxford University's Blavatnik School of Government [8, 35]. For the purpose of this study, we have also cross-referenced country-specific sources such as the Ministry of Health of Israel.

#### 2.1.2. Google COVID-19 Community Mobility Reports

We have also utilized the Google COVID-19 Community Mobility Reports (GCMR) which uses aggregated and anonymized data to chart changes in mobility with respect to different classes of places—retail and recreation, groceries and pharmacies, parks, transit, workplace, and residential (https://www.google.com/covid19/mobility/). Mobility indicators are calculated based on the frequency and length of visits to places.

The data used in this study were extracted from the Global COVID-19 Index and GCMR on the 5^th^ of each month from July to December 2021. Countries selected for inclusion are among the earliest countries to have started vaccination of its population against COVID-19, i.e., the United Kingdom (GBR), United States of America (USA), Israel (ISR), Canada (CAN), France (FRA), Italy (ITA), and Austria (AUT). These countries also share similar characteristics in terms of income level and age structures. Additional was the criterion that complete data is needed to be available for new case numbers, deaths, hospitalisations, ICU admissions, and vaccinations (full and partial) due to COVID-19. Hence, the dataset consists of new cases, hospitalisations, ICU admissions and deaths due to the virus, stringency of government responses to the pandemic, partial (single dose) and full (double doses) vaccinations, and changes in human mobility across the six different classes of places.

New cases, number of hospitalisations, number of ICU admissions, and number of deaths due to COVID-19 collectively indicate the spread of the virus and the severity in the country. Partial and full vaccinations indicate the proportion of each country's population having received single or double doses of COVID-19 vaccines, respectively. The stringency measure is an indicator which reflects the stringency of government responses to the COVID-19 pandemic, with lower values also implying the easing of restrictions within the country. The six different mobility indicators from GCMR reflect the changes in mobility in each class of places—retail and recreation, groceries and pharmacies, parks, transit, workplace, and residential.

### 2.2. Data Analysis

Principal component analysis (PCA) was employed in this study. PCA is a robust multivariate technique for dimensionality reduction and data visualization [36, 37]. Supposing that there are *n* observations with measurements on a set of *p* variables, this technique uses dependencies between variables (*X*_1_, *X*_2_, ⋯, *X*_*p*_) to produce a low-dimensional representation of a dataset, while preserving as much information as possible. The PCA technique derives the principal components by finding a sequence of linear combinations of the variables that have maximal variance and are mutually uncorrelated [36]. The first principal component, *Z*_1_, accounts for the maximal amount of the total variance of the observed variables. The second component, *Z*_2_, is orthogonal to the first component and will account for the maximal amount of variance in the dataset that was not accounted for by the first component. Likewise, the third principal component, *Z*_3_, is the direction which maximizes variance among all directions, not accounted for by the first and second components.

The first three principal components are given by the following formulae:

(1) $$\begin{array}{c} Z_{1}=\phi_{11}X_{1}+\phi_{21}X_{2}+\cdots +\phi_{p1}X_{p},\\ Z_{2}=\phi_{12}X_{1}+\phi_{22}X_{2}+\cdots +\phi_{p2}X_{p},\\ Z_{3}=\phi_{13}X_{1}+\phi_{23}X_{2}+\cdots +\phi_{p3}X_{p}. \end{array}$$

PCA is based on the decomposition of the original data matrix into the scores and loading matrices. The scores (e.g., for *Z*_1_: *z*_11_, ⋯, *z*_*n*1_) classify the samples, whereas loadings (e.g., for *ϕ*_1_: *ϕ*_11_, ⋯, *ϕ*_*p*1_) classify the variables and are referred to as the weight for each variable [36]. More details about the PCA technique can be found elsewhere [36, 37].

The PCA technique is suitable for achieving the research objective of this study. This technique examines multiple variables in a multivariate context, reducing the dimensionality of the data and improving interpretability while preserving as much information as possible. Other researchers have also used this technique as a dimensionality reduction and feature extraction tool in various research areas [38–41].

We first presented the summary statistics on new cases, hospitalisations, ICU admissions, and deaths due to COVID-19, stringency of government responses to the pandemic, and partial and full vaccinations. We computed the correlation matrix on these variables and Google mobility indicators, then followed by principal component analyses. The PCA technique was performed using the “prcomp” command of the R statistical software, after standardizing each variable to have mean zero and standard deviation of one. Standardization involves rescaling the variables such that each will have the properties of a standard normal distribution with a mean of zero and a standard deviation of one. The principal component score vectors were extracted from the results and visualized through 2-dimensional and 3-dimensional plots using the “ggplot2” and “scatterplot3d” R packages [42, 43].

## 3. Results

Summary statistics from data extracted on the 5^th^ of each month from July to December 2021, are presented in Table 1 grouped by the seven respective countries. During this period, the numbers of new cases, hospitalisations, ICU admissions, and deaths were on average higher in the USA compared to the other countries. By 5^th^ December 2021, all seven countries have administered full COVID-19 vaccine to at least 60% of its population. The proportion for the USA is comparatively the lowest at 60%, whereas Canada achieved the highest at 76%.

The average stringency levels in Canada is also comparatively higher than those in the other six countries, as its stringency index hovers between 57 and 72 throughout this period. The UK government's stringency measures have hovered around average levels (41–51). There is a similar pattern in the stringency levels for Austria, France, Italy, and Israel as its stringency values have increased gradually (not shown here) from 5^th^ July to 5^th^ December 2021. Oddly, the stringency in the USA has decreased from 62 on the 5^th^ of July 2021, to the value of 48 on the 5^th^ of December 2021 (not shown here).

The correlation matrix is presented in Table 2. New cases, hospitalizations, ICU admissions, and deaths due to COVID-19 are strongly positively correlated with each other (0.80–1.0). Full and partial vaccinations show strong (0.80–1.0) positive correlation with each other and moderate (0.40–0.59) positive association with stringency measures. Notably, full vaccination is moderately negatively correlated (0.40–0.59) with park mobility, but weaker (<0.4) with other variables such as new cases, hospitalisations, and retail and recreation mobility.

On the other hand, the strength and direction of correlation between mobility indicators vary. Residential mobility is negatively correlated with all other mobility indicators. In terms of strength, it has a strong association (>0.60) with transit and workplace-related mobility, but moderate association (0.40–0.59) with retail and recreation and grocery and pharmacy-related mobility. Correlations also show that transit and workplace mobility are strongly associated with each other, similarly to the association between retail and recreation and groceries and pharmacies. Park mobility has a moderate association (0.40–0.59) with both retail and recreation and grocery and pharmacy mobility.

Table 3 displays the proportion of variance explained by all of the principal components. As there are 13 variables in the analysis, 13 principal components (PC1, PC2, PC3,…, PC13) are produced. The variance of the data contributed by the first (PC1), second (PC2), and third (PC3) principal components is around 31.6%, 24.3%, and 21.4%, respectively. Taken together, the first three components can explain around 77.3% of the variance in the data. Of the remaining variance, the fourth component captures around 8.5%, whereas the rest captures less than 5% each.

The principal component loading vectors are presented in Table 4. The first loading vector places rather large, positive, and approximately equal weights on confirmed cases, hospital and ICU admissions, and deaths due to the virus. The weights for full and partial vaccinations, stringency, and mobility indicators are relatively smaller. Hence, this first component seems to clearly correspond towards an indication of the level of severity of the COVID-19 surge in the country. Countries with large positive scores on the first component would indicate high severity levels.

In the second loading vector, the weights of retail and recreation, grocery and pharmacy, transit, and residential mobility indicators are relatively large compared to the rest of the variables. These indicators have negative projections, except for residential mobility. Therefore, the second component appears to reflect more on human mobility patterns, where countries with large negative scores would indicate more of transit, retail, and recreational types of mobility.

The third loading vector places rather large, positive, and approximately equal weights on workplace mobility and full and partial vaccinations. Hence, countries with large positive scores on this component would indicate higher vaccination coverage and workplace mobility. The weights for stringency measures and residential mobility (with opposing sign) are slightly smaller and less dominant in this third loading vector. Hence, countries with large positive scores would also indicate, albeit to a lesser degree, higher government stringency measures and less mobility trends for places of residences. The weights for workplace and residential mobility are rather intuitive, as there is a strong negative correlation between these indicators.

In the fourth loading vector, the weights of stringency measures and parks are negative and comparatively larger. Hence, countries with large negative scores imply more stringent measures imposed by its government, as well as higher park-related mobility. However, this principal component contributes only 8.5% of the variance in the data.

**Figure 1:**
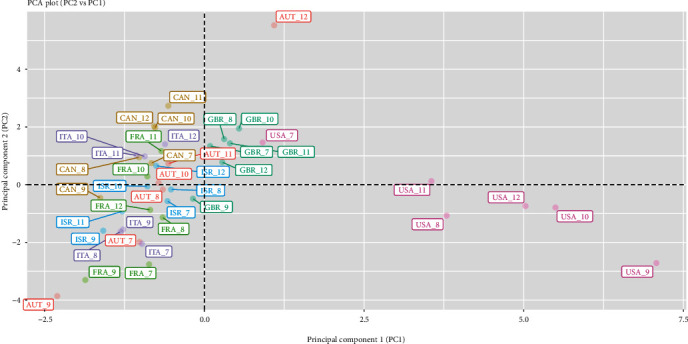
PCA plot (second principal component, PC2, vs. first principal component, PC1).

**Figure 2:**
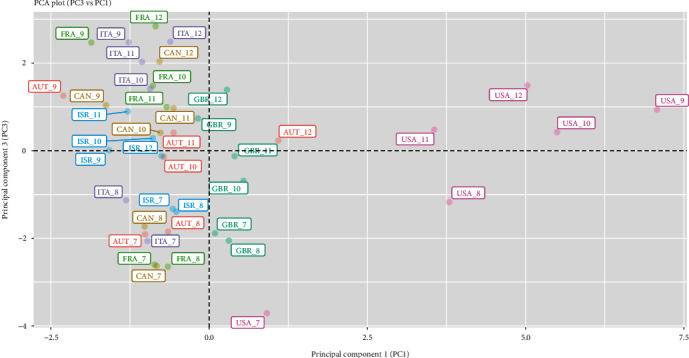
PCA plot (third principal component, PC3, vs. first principal component, PC1).

The first component is driven by the spread of COVID-19, the second component by mobility activities (transit, retail, and recreational), whereas the third by COVID-19 vaccination coverage, workplace-related mobility, and government stringency measures. As these three principal components contribute to a substantial amount of variance in the data (77.3%), we have presented them in plots to further aid in interpretation. We can examine differences between the seven countries via two 2-dimensional plots of three principal component score vectors as shown in Figures 1 and 2. Each country is displayed as coloured text, labelled according to the country's ISO code and month, and represents the scores of the principal components in the corresponding plots.

On the first principal component (PC1) (Figure 1), Austria, Canada, France, the UK, Israel, and Italy appear either near the origin (0, 0) or on the left of the PC1 axis, indicating moderate or lower severity in the COVID-19 pandemic between July and December 2021. Though Austria appears to be facing a higher surge in December. By contrast, the USA has large positive scores on the first component, indicating especially a severe surge in the month of September.

On the second principal component (PC2) (Figure 1), Austria, Canada, France, Italy, and Israel as captured in September have larger negative scores on PC2, reflecting higher transit, retail, and recreational-related mobility during that month. Coincidently, the USA appears on the bottom right quadrant in the plot indicating very high mobility behaviour and a severe COVID-19 surge concurrently in September. Interestingly, mobility patterns in Austria as seen in September are starkly different from those in December.

The third principal component (PC3) (Figure 2) indicates clearly the rollout in full and partial vaccinations, as well as workplace mobility. It is clearly seen that from July to December 2021, all seven countries have increased vaccination coverage of its population and workplace-related mobility has also increased. PC3 also somewhat indicates more stringent measures imposed by governments. In December 2021, France, Italy, and Canada appear at the top of PC3, indicating high vaccination coverage of its population, as well as stricter government response to the pandemic. As these countries appear to the left of the PC1 axis, it appears that the COVID-19 situation in these countries is under good control. It is also notable that high vaccination coverage together with moderate levels of government stringency appears adequate in controlling the spread of COVID-19, as seen in Israel and the UK. The spread of COVID-19 is more severe in the USA, although the country's stringency is at a moderate level. The country's stringency level is rather similar to the UK in December, but its full vaccination coverage of its population is the lowest compared to the other six countries in the study.

**Figure 3:**
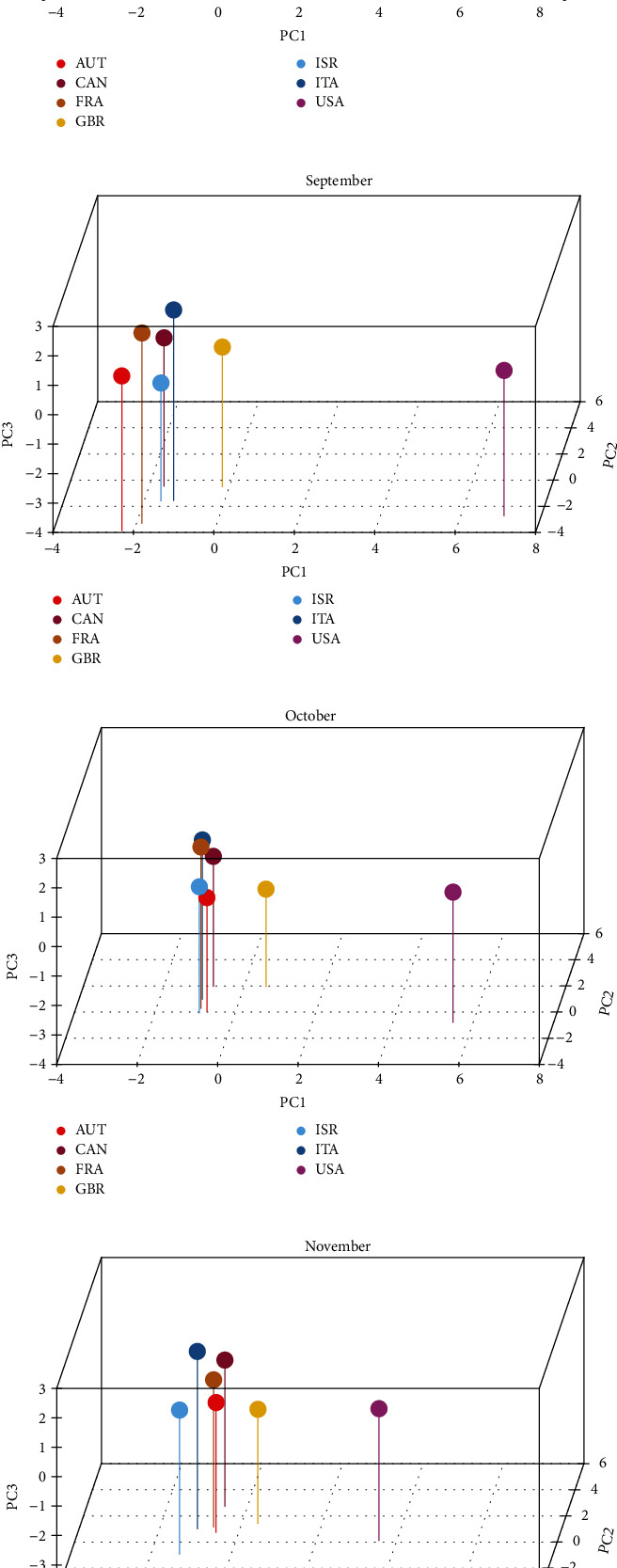


The 3-dimensional plots (Figures 3(a)–3(f)) of the seven countries reveal the patterns in the data captured through the three principal components, according to months. Throughout the 6 months, it can be seen that Austria, Canada, France, Italy, and the UK are somewhat clustered together and have rather similar patterns in terms of human mobility behaviour, vaccination coverage, government stringency measures, and overall control of the pandemic. On the other hand, the USA appears to have had a somewhat similar experience in July 2021. However, as the country continued to ease restrictions since July, its initial control of the pandemic has diverged beginning August 2021, although vaccine coverage increased.

## 4. Discussion

The order of principal components reflects the amount of variance captured from the data. Hence, it is evident that this data of seven countries, captured between 5^th^ July and 5^th^ December 2021, primarily explains the severity of the COVID-19 pandemic, followed by transit, retail, and recreational-related mobility patterns, then followed by the vaccination coverage, workplace-related mobility, and government stringency measures. During this period, the Delta variant has spread widely in these countries and has more than doubled the risk of hospital admissions and death compared to the Alpha variant [24, 27]. However, with widespread vaccine coverage and at least moderate levels of government stringency measures, our findings indicate that the COVID-19 pandemic has largely been contained in Austria, France, Italy, Israel, Canada, and the UK.

It is also apparent that the pandemic and accompanying government regulations and restrictions have influenced human mobility behaviour [23]. Our findings reveal changes in human mobility patterns in these seven countries during the second half of 2021, considering the widespread vaccination rollout and rather moderate levels of government stringency measures and the spread of COVID-19 simultaneously. Human mobility activities peaked in September for most countries, particularly with regard to transit, retail, recreation, groceries, and pharmacy types of mobility. These activities then receded in the following months as countries began to grapple with a surge in COVID-19 cases and severity, and governments such as France and Italy have imposed more stringent measures. The striking change in human mobility patterns in Austria in December of 2021 coincides with the country's stringent measures to curb the surge in cases. Austria had imposed widespread restrictions and entered full national lockdown during the end of November 2021 [44].

The COVID-19 pandemic in the USA appears to have progressively worsen after July of 2021. Although vaccination coverage of its population has increased, the government stringency levels have continued to decrease. The country's continued easing of restrictions may have contributed to the resurgence of infections. The surge in the pandemic peaked in September, in tandem with the peak in human mobility behaviour. Besides the less stringent government measures and increase in human mobility, there are other potential extenuating factors which may have exacerbated the surge between August and December 2021. The cases in the USA have increased due to the combination of the Delta variant's easier transmissibility and Americans using less mask and practicing less social distancing [27]. Vaccine coverage also depends on concurrent adherence to interventions such as wearing of masks and physical distancing [45]. Despite less severe infections among patients due to vaccine protection, the impact by sheer numbers itself may have intensified pressure on hospitals already strained by the pandemic [46]. Moreover, although the USA and Europe population age structures are rather similar, the former's population is comparatively less healthy than the latter. The prevalence of hypertension and obesity is higher in the USA than in most other countries, and these conditions lead to higher risks of severe illness from COVID-19 [46].

In our findings, higher vaccination coverage coincides with increased workplace-related mobility, indicating increasing economic activities at workplaces. Workplace and other retail and recreational-related mobility behaviours during this period implies the recovery of labour markets during the second half of 2021. By contrast, park-related mobility is not as dominant compared to other types of mobility, unlike what was observed after the first prolonged lockdown due to the COVID-19 pandemic in 2020 [47]. From what we have gleaned from the fourth principal component, higher park-related mobility would coincide with more stringent government measures. However, government stringency measures in most of these seven countries have been at rather moderate levels in the middle of 2021, though, as the individual stringency values would indicate, stringency measures in France, Italy, and Austria have increased by early December 2021.

Our findings indicate that the rate of vaccination coverage is important. Mass COVID-19 vaccinations work together with at least moderate levels of government stringency measures in order to control the spread of COVID-19, as seen in Austria, Italy, France, Israel, Canada, and the UK. Some of these countries have also started the COVID-19 vaccine booster programmes in July of 2021 and progressively administered vaccine boosters to their population [16, 30]. The most notable countries were Israel, the UK, and Austria, where around 30–40% of their population have had a booster vaccine by early December 2021 [9]. Towards the end of 2021, many other countries in North America and Europe have also expanded their COVID-19 vaccine booster programmes and expanded eligibility to all fully vaccinated adults [48].

COVID-19 vaccine protection through widespread vaccination rollout is essential to ensure that hospitals are not overwhelmed with severe cases, to revive economic activities and labour markets, and to allow the return to some semblance of normalcy. However, the public's perception, belief, and attitudes towards the COVID-19 vaccine have undue influence on the rate of vaccine uptake [49]. Culture and other macroenvironmental factors can also influence a nation's vulnerability and effectiveness in handling the pandemic [50]. Moreover, paranoia and widespread misinformation will continue to impede the attainment of herd immunity and recovery from the COVID-19 pandemic [51, 52]. Hence, governments need to proactively engage with the public on the importance and safety of vaccination, and for some nations, the communication needs to be culturally sensitive and inclusive to all religious societies [50, 53]. Effective communication through media and news agencies is imperative to dispel fears, mental anxieties, and battle of COVID-19 infodemics [51].

It is also necessary to continuously assess risks and adjust countermeasures. We believe that these findings reveal the importance of governments establishing strong data collection mechanisms that can track and categorise severity of symptoms, hospitalisations, and ICU admissions. These will enable mathematical models to be applied to provide real-time advisory to policy-makers on appropriate responses and time frames, of which measures can either be loosened or even tightened in light of what the models are able to forecast.

## 5. Conclusions

This study utilises PCA, a multivariate unsupervised learning technique, to derive deeper insights into the control of the spread of COVID-19, during the period of 5^th^ July to 5^th^ December 2021, from seven of the earliest vaccinated countries. We have delineated these dimensions, the spread of COVID-19, human mobility patterns, widespread vaccination rollout, and government stringency measures, on the overall control of the COVID-19 pandemic. Another novelty from this study is the utilisation of this multivariate technique as a visualization tool to reveal hidden patterns in the data during this period when the highly transmissible COVID-19 Delta strain was dominant.

The key findings from this study are summarized as below:

- The variance in the data related to these seven countries during this period reveals that the spread of COVID-19 was most dominant. Slightly less dominant were the human mobility patterns, the widespread vaccination rollout, and government stringency measures, respectively
- PCA visualizations indicate comparatively lower or moderate levels of severity in the COVID-19 pandemic between July and December 2021, in Austria, Italy, France, Israel, Canada, and the UK. By contrast, the USA faced more severe COVID-19 outbreaks especially in September of 2021
- Human mobility activities peaked in September for most countries and then receded in the following months as more stringent government measures were imposed, and countries began to grapple with a surge in COVID-19 cases
- All seven countries have increased vaccination coverage of their population, and workplace-related mobility has also increased during this period
- Widespread vaccination rollout is particularly important. High vaccination coverage, together with at least moderate levels of government stringency measures, is able to control the spread of COVID-19, as seen in Austria, Italy, France, Israel, Canada, and the UK

There are limitations in the study. Here, the PCA technique is applied solely for exploratory data analysis. As PCA is often viewed as a dimensionality reduction technique, the model complexity is determined in terms of the number of principal components that explain most of the systematic variation in the data [36, 37, 54]. The first three principal components were extracted, visualized, and interpreted through loading vectors and visualizations. These three main components account for around three-fourths of the variance in the data. The remaining components, each, captured less than 10% of the variance. Hence, they were not visualized and discussed in our study. However, we argue that the first three components are highly representative of the variance in the data and can clearly delineate the different dimensions which are most dominant.

Moreover, due to lack of COVID-19 vaccination coverage and incomplete and insufficient data on ICU and hospital admissions in other countries, only seven countries were included in the multivariate analysis. Data from more countries, captured on shorter time intervals, may provide for a deeper analysis. For future research, the waning effects of vaccination and other confounding factors at play during the Omicron wave may deserve apt attention, when more data becomes available. Our findings provide suggestions for governments globally to consider and serve as a reference when the next pandemic of highly infectious disease comes along.

## Figures and Tables

**Table 1:** Summary statistics by country (data extracted on the 5^th^ of each month from July to December 2021).

	Mean	Std deviation	Min	Max
*Austria (AUT)*				
New cases	3063	3324	81	7885
Hospitalisations	848	919	83	2402
ICU admissions	249	233	38	654
Deaths	15	22	1	58
Full vaccination (%)	57%	10%	38%	67%
Partial vaccination (%)	63%	6%	55%	71%
Stringency index	56	7	49	68

*Canada (CAN)*				
New cases	2382	1421	485	3943
Hospitalisations	1390	755	480	2522
ICU admissions	471	187	200	759
Deaths	21	12	9	43
Full vaccination (%)	65%	15%	36%	76%
Partial vaccination (%)	75%	5%	68%	81%
Stringency index	67	5	57	72

*France (FRA)*				
New cases	15362	15189	2353	42596
Hospitalisations	8670	1961	6735	11526
ICU admissions	1525	497	1077	2217
Deaths	61	34	24	111
Full vaccination (%)	58%	14%	34%	70%
Partial vaccination (%)	70%	10%	52%	77%
Stringency index	60	12	44	72

*United Kingdom (GBR)*				
New cases	34066	7542	25995	45849
Hospitalisations	6522	2310	2364	9091
ICU admissions	828	241	369	1026
Deaths	103	51	18	172
Full vaccination (%)	62%	7%	50%	68%
Partial vaccination (%)	71%	3%	67%	75%
Stringency index	46	3	41	51

*Israel (ISR)*				
New cases	2897	3427	338	9427
Hospitalisations	562	484	101	1346
ICU admissions	120	87	15	240
Deaths	11	13	0	31
Full vaccination (%)	60%	2%	56%	62%
Partial vaccination (%)	65%	3%	61%	69%
Stringency index	48	13	30	56

*Italy (ITA)*				
New cases	5765	4676	778	14466
Hospitalisations	3708	1673	1528	6333
ICU admissions	433	199	191	736
Deaths	44	21	19	74
Full vaccination (%)	61%	15%	34%	73%
Partial vaccination (%)	71%	8%	59%	79%
Stringency index	62	11	47	71

*United States of America (USA)*				
New cases	95830	51142	12226	165263
Hospitalisations	54731	26984	13433	95279
ICU admissions	14741	7224	3883	25630
Deaths	1147	672	193	1814
Full vaccination (%)	54%	4%	48%	60%
Partial vaccination (%)	64%	6%	56%	71%
Stringency index	54	5	48	62

**Table 2:** Correlation matrix.

	New cases	Hosp.	ICU	Deaths	Full Vac.	Partial Vac.	Stringency	Retail & recreational	Grocery & pharmacies	Parks	Transit	Workplace	Residence
New cases	1	0.96	0.94	0.89	-0.06	-0.06	-0.2	-0.06	-0.29	-0.23	-0.03	0.03	0
Hosp.	0.96	1	1	0.91	-0.13	-0.15	-0.11	-0.02	-0.26	-0.17	0.02	0	-0.02
ICU	0.94	1	1	0.92	-0.14	-0.18	-0.09	-0.02	-0.26	-0.16	0	-0.02	-0.01
Deaths	0.89	0.91	0.92	1	-0.08	-0.09	-0.11	-0.06	-0.3	-0.24	-0.03	0.03	-0.01
Full Vac.	-0.06	-0.13	-0.14	-0.08	1	0.85	0.44	-0.24	-0.18	-0.52	-0.07	0.39	-0.04
Partial Vac.	-0.06	-0.15	-0.18	-0.09	0.85	1	0.58	-0.2	-0.14	-0.33	-0.21	0.37	0
Stringency	-0.2	-0.11	-0.09	-0.11	0.44	0.58	1	-0.09	0.08	-0.1	-0.05	0.24	-0.06
Retail & recreational	-0.06	-0.02	-0.02	-0.06	-0.24	-0.2	-0.09	1	0.72	0.51	0.41	0.18	-0.44
Grocery & pharmacies	-0.29	-0.26	-0.26	-0.3	-0.18	-0.14	0.08	0.72	1	0.4	0.56	0.22	-0.42
Parks	-0.23	-0.17	-0.16	-0.24	-0.52	-0.33	-0.1	0.51	0.4	1	0.02	-0.18	-0.16
Transit	-0.03	0.02	0	-0.03	-0.07	-0.21	-0.05	0.41	0.56	0.02	1	0.61	-0.75
Workplace	0.03	0	-0.02	0.03	0.39	0.37	0.24	0.18	0.22	-0.18	0.61	1	-0.86
Residence	0	-0.02	-0.01	-0.01	-0.04	0	-0.06	-0.44	-0.42	-0.16	-0.75	-0.86	1

**Table 3:** Proportion of variance explained from PCA analysis.

	PC1	PC2	PC3	PC4	PC5	PC6	PC7	PC8	PC9	PC10	PC11	PC12	PC13
Proportion of variance	0.316	0.243	0.214	0.085	0.047	0.044	0.017	0.014	0.009	0.008	0.003	0.002	0.000
Cumulative proportion	0.316	0.558	0.773	0.858	0.905	0.949	0.966	0.979	0.988	0.995	0.998	1.000	1.000

**Table 4:** Principal component loading vectors from PCA analysis.

	PC1	PC2	PC3	PC4	PC5	PC6	PC7	PC8	PC9	PC10	PC11	PC12	PC13
New cases	0.462	-0.112	0.091	-0.069	0.083	-0.133	0.179	-0.19	0.274	0.278	-0.057	-0.706	-0.117
Hosp.	0.464	-0.146	0.07	-0.126	0	0.057	0.116	0.084	0.257	0.078	0.075	0.35	0.72
ICU	0.465	-0.142	0.059	-0.14	-0.026	0.084	0.038	0.118	0.208	-0.1	0.042	0.456	-0.674
Deaths	0.454	-0.108	0.093	-0.089	-0.004	0.006	-0.131	-0.094	-0.773	-0.34	-0.094	-0.112	0.069
Full Vac.	-0.054	0.274	0.456	-0.066	0.302	-0.254	0.255	0.448	0.141	-0.475	-0.172	-0.111	0.035
Partial Vac.	-0.073	0.285	0.428	-0.304	0.077	-0.311	0.185	-0.252	-0.272	0.439	0.342	0.224	-0.061
Stringency	-0.102	0.153	0.317	-0.518	-0.336	0.629	-0.17	0.122	0.059	0.032	-0.041	-0.197	0.014
Retail & recreational	-0.127	-0.417	-0.024	-0.394	0.364	-0.27	-0.554	0.316	-0.002	0.201	-0.033	-0.033	0.002
Grocery & pharmacies	-0.25	-0.367	0.022	-0.279	0.441	0.247	0.282	-0.532	0.086	-0.309	-0.011	0.056	0.01
Parks	-0.154	-0.253	-0.286	-0.426	-0.497	-0.344	0.462	0.146	-0.091	-0.045	-0.197	-0.009	-0.001
Transit	-0.103	-0.418	0.223	0.31	0.136	0.334	0.399	0.403	-0.286	0.365	-0.02	-0.014	-0.052
Workplace	-0.083	-0.216	0.494	0.211	-0.259	-0.167	-0.19	-0.298	0.098	0.087	-0.625	0.176	0.012
Residence	0.103	0.401	-0.327	-0.177	0.351	0.155	0.116	0.012	-0.115	0.304	-0.634	0.146	-0.013

## Data Availability

We have acquired access to data from the Global COVID-19 Index (GCI) provided by PEMANDU Associates. The GCI is an independent data engine covering 180 economies and consolidates to provide open data (https://covid19.pemandu.org/). We also utilized the Google COVID-19 Community Mobility Reports (GCMR) which uses aggregated and anonymized data to chart changes in mobility with respect to different classes of places—retail and recreation, groceries and pharmacies, parks, transit, workplace, and residential (https://www.google.com/covid19/mobility/).
